# Functional Nanocomposites: Preparation, Characterization and Applications

**DOI:** 10.3390/ma19143021

**Published:** 2026-07-13

**Authors:** Małgorzata Wasilewska, Dariusz Sternik

**Affiliations:** Department of Physical Chemistry, Faculty of Chemistry, Institute of Chemical Sciences, Maria Curie-Sklodowska University, Maria Curie-Sklodowska Sq. 3, 20-031 Lublin, Poland; dariusz.sternik@mail.umcs.pl

## 1. Introduction and Scope

Functional nanocomposites are currently one of the most dynamically developing groups of materials, combining the properties of various components at the nano- and micrometric level to achieve new functionalities unavailable to conventional materials [1,2]. Advances in synthesis, surface modification, material architecture design, and advanced characterization methods enable the creation of systems with precisely controlled physical, chemical, and biological properties [3]. A particularly well-studied example is polymer–silica nanocomposites, in which sol–gel methods, in situ polymerization and advanced surface functionalization allow for significant improvement of mechanical, thermal and barrier properties [4].

Growing demands related to environmental protection, energy storage, water purification, circular economy, and the development of modern technologies are making functional nanocomposites the subject of intensive research and implementation [5]. Designing materials based on renewable raw materials, biocomponents, and carbon and magnetic nanomaterials currently plays a particularly important role, combining high performance with the principles of sustainable development [6,7,8]. Examples include magnetic MnFe_2_O_4_ nanocomposites with carbon dots enabling simultaneous adsorption and detection of heavy metals (e.g., U(VI)) [9], as well as chitosan–graphene oxide systems dedicated to selective removal of Cr(VI) from aqueous solutions [10]. In the field of energy storage, mesoporous carbon composites with graphene or carbon nanotubes are being intensively developed, constituting high-quality electrode materials for supercapacitors and electrocatalysis due to their hierarchical porosity and excellent conductivity [11]. Additionally, increasing emphasis is being placed on sustainable nanocomposites based on lignin or biopolymers, which are used in pollutant adsorption and biomedicine [12,13]. A broad review of functional nanocomposites and their potential applications is presented by Hassan et al. [14].

The aim of this Special Issue was to gather papers presenting the latest advances in the preparation, characterization, and applications of functional nanocomposites. The published articles include both experimental studies and review articles. They present modern adsorption materials designed to remove pollutants from water [2], magnetic nanocomposites, advanced carbon materials for electrochemical applications, biocomposites developed in accordance with the principles of green chemistry [7], and issues related to assessing the recyclability of composite materials.

A common denominator of all these publications is the exploration of the relationships between the structure, surface properties, and functionality of materials, as well as the development of solutions enabling the effective use of nanocomposites in practical environmental and technological applications [1,5,15].

## 2. An Overview of the Published Articles

The first published work concerns the development of biocomposite adsorbents based on sodium alginate, activated carbon, and cellulose for the removal of nonsteroidal anti-inflammatory drugs from aquatic environments [16]. The authors demonstrated that the appropriate selection of composite components significantly increased the material’s specific surface area and improved its adsorption capacity. Particularly high efficiency was achieved for systems containing activated carbon, confirming the significant role of the developed porous structure and surface properties in the removal of pharmaceutical micropollutants from water.

The issue of aquatic environment purification was further explored in a review article devoted to the use of nanomaterials in chromium removal and speciation processes [17]. The authors presented a broad overview of contemporary adsorption materials, including carbon nanotubes, graphene materials, magnetic nanocomposites, metal oxides, and layered double hydroxides. Particular attention was paid to the importance of surface functionalization and the design of active sites enabling the selective removal of various chemical forms of chromium. The paper highlights the importance of nanotechnology in monitoring and eliminating one of the most dangerous environmental pollutants.

Energy storage and electrochemistry also feature prominently in this Special Issue. A review article on mesoporous carbon composites containing carbon nanostructures presented the current state of knowledge on materials based on graphene, carbon nanotubes, and carbon nanoonions. The authors demonstrated that appropriate shaping of the porous structure, control of electrical conductivity, and chemical modifications enable the production of high-performance materials in applications such as supercapacitors, electrocatalysis, and energy storage. The article outlines directions for the further development of advanced carbon nanocomposites used in modern energy technologies [18].

A significant portion of this Special Issue consists of papers devoted to adsorbents for the treatment of wastewater and water contaminated with organic substances. Ramos-Guivar and colleagues developed a biofunctional magnetic nanohybrid carbon material composed of multi-walled carbon nanotubes and maghemite nanoparticles obtained using an extract from Eucalyptus globulus [19]. The resulting material demonstrated high adsorption capacity for methylene blue, with the added advantage of easy recovery of the adsorbent from the solution using a magnetic field. The authors also demonstrated the material’s high stability and the possibility of repeated use without significant loss of adsorption properties.

The next paper focused on the use of biochar obtained from tomato waste as a matrix for the production of composites containing Fe_3_O_4_ and MnO_2_ oxides [20]. The authors proposed an interesting solution that aligns with the principles of a circular economy by utilizing agricultural waste for the production of high-performance adsorption materials. The resulting composites demonstrated high efficiency in removing paracetamol from aqueous solutions, and studies of adsorption isotherms, kinetics, and thermodynamic parameters confirmed the favorable course of the adsorption process. A particularly important aspect of the work was the combination of the biochar’s adsorption properties with the functionality of oxide nanoparticles.

The last article presents a different but extremely important issue related to the evaluation of composite material recycling. The authors developed a methodology for quantitatively assessing the recovery efficiency of particles from paraffin–tungsten composites using optical microscopy and digital image processing techniques [21]. The proposed approach enabled quantitative analysis of particle distribution, surface area, and density before and after the recycling process. The results clearly confirmed the effectiveness of the developed recovery procedure and highlighted the growing importance of designing materials with their full life cycle in mind.

Collectively, the presented works demonstrate a broad spectrum of research on functional nanocomposites—from the design of new materials, through advanced structural and surface characterization, to environmental, energy, and technological applications. A particularly visible trend is the use of biodegradable materials, biomass waste, carbon nanomaterials, and magnetic components to achieve high process efficiency while reducing environmental impact [18,19].

## 3. Conclusions

The articles collected in this Special Issue confirm that functional nanocomposites remain one of the most important development directions in contemporary materials science. The studies presented demonstrate that appropriate design of material structures at the nano- and micrometric level allows for the necessary properties to be obtained to solve current technological and environmental problems.

A significant portion of the published works focused on issues related to environmental protection, particularly the purification of pharmaceuticals, dyes, and heavy metals from water. At the same time, the growing importance of materials based on renewable raw materials, biomass waste, and carbon nanomaterials was demonstrated. The papers included in this Special Issue also highlight the need to consider issues related to material recovery and recycling at the design stage.

The collected publications demonstrate that the future development of functional nanocomposites will be based on the integration of advanced synthesis methods, multi-technique material characterization, and a sustainable development approach. We hope that the results presented in this Special Issue will inspire further research and contribute to the development of innovative materials for environmental protection, energy, and modern technologies.
